# Duplication of the Gallbladder as an Operative Surprise: A Case Report with Review of the Literature

**DOI:** 10.1155/2021/6668302

**Published:** 2021-02-11

**Authors:** Jai P. Singh

**Affiliations:** Department of Surgery, Oswego Hospital, New York 13126, USA

## Abstract

**Introduction:**

Duplication of the gallbladder is a rare congenital anomaly of the biliary system. Anomalous anatomy of the biliary system is associated with an increased risk of complications such as bile duct injury during cholecystectomy. Herein, I present a case report of duplication of the gallbladder, which was an operative surprise as the patient's preoperative workup did not reveal any evidence of duplication of the gallbladder. *Case Report*. A 60-year-old female was admitted for management of recurrent pancreatitis. Diagnosis of biliary pancreatitis was made as her CT and US revealed cholelithiasis. During laparoscopic cholecystectomy, she was found to have duplication of the gallbladder, which was a surgical surprise. Both the gallbladders were successfully removed, and the patient had an uneventful postoperative course.

**Conclusion:**

Duplication of the gallbladder is a rare congenital anomaly, which could be associated with other congenital anomalies of the bile duct and vascular system. Extreme care should be taken during cholecystectomy as these anomalies could lead to serious injury to the bile duct and vessels.

## 1. Introduction

Gallbladder duplication is a rare congenital malformation seen in around one in 3800-4000 births [1]. The exact incidence cannot be ascertained as most cases are detected at autopsy, surgery, or imaging [1]. The congenital malformations are considered the predisposing risk factors for iatrogenic bile duct injury. Herein, I present a case report of duplication of the gallbladder, which was an operative surprise as the patient's preoperative workup did not reveal any evidence of duplication of the gallbladder.

## 2. Case Report

**Figure 1:**
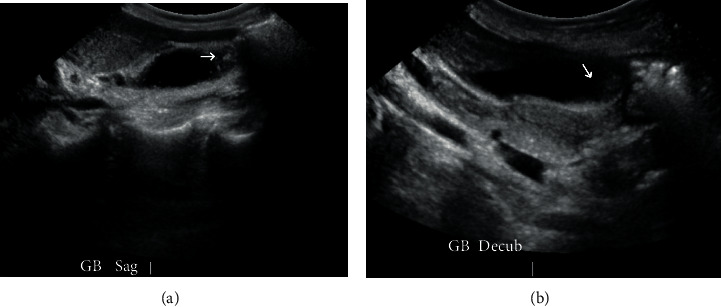
(a) US of the gallbladder, sagittal view revealed cholelithiasis. (b) US of the gallbladder, decubitus view revealed cholelithiasis.

**Figure 2:**
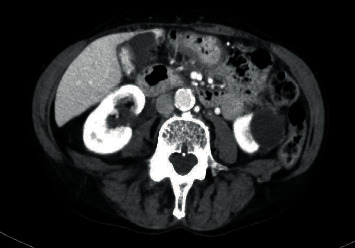
CT abdomen reported cholelithiasis with pericholecystic fluid.

**Figure 3:**
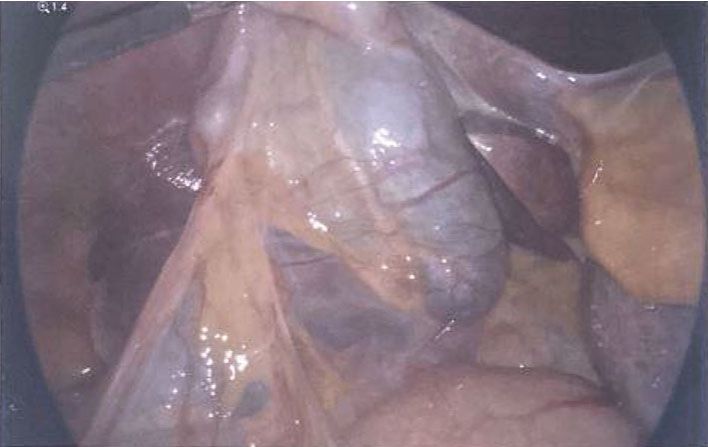
Intraoperative picture with one peritoneal lining.

**Figure 4:**
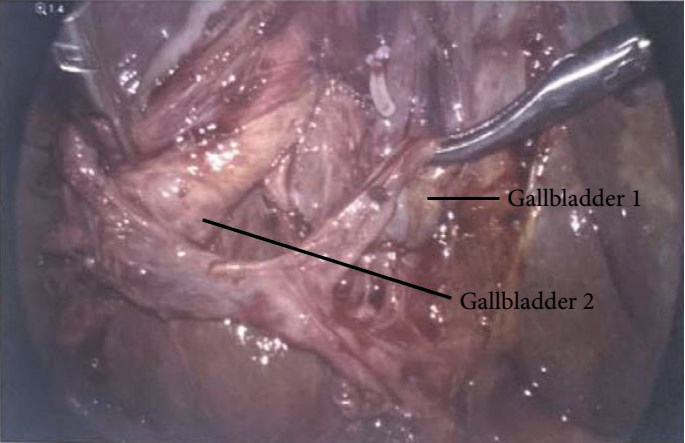
Two separate gallbladders after incomplete cystic duct dissection.

**Figure 5:**
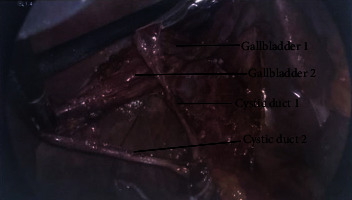
Two separate gallbladders with two separate cystic ducts after further dissection.

**Figure 6:**
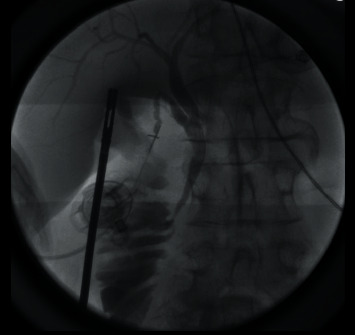
Intraoperative cholangiogram revealed single channel entering common bile duct.

A 60-year-old female presented to the emergency room with a sudden onset of abdominal pain in the epigastrium for one day. The pain was severe and sharp and was radiating to the back. She also had some nausea but no vomiting. She had two episodes of acute pancreatitis in the past. Her history was also significant for chronic bronchitis, congestive heart failure, hypertension, idiopathic thrombocytopenia, and osteoarthritis. Her labs revealed white blood count (WBC) 15700/*μ*L, platelet 97,000/*μ*L, and lipase 219 U/L. All other labs, including liver function tests, were unremarkable. Her ultrasound revealed cholelithiasis without cholecystitis with a nondilated bile duct (Figures 1(a) and 1(b)). Computed tomography (CT) abdomen reported cholelithiasis with pericholecystic fluid (Figure 2). CT scan did not reveal any inflammatory changes in the pancreas. Diagnosis of recurrent pancreatitis was made based on her clinical presentation and labs. Because of cholelithiasis on the ultrasound and CT scan, a diagnosis of gallstone pancreatitis was made. The patient was admitted to the hospital for pain control, bowel rest, and IV fluids. The next day, her pain was much better, and her lipase dropped to 56. After proper optimization of her medical comorbidities, the patient was brought to the operating room for cholecystectomy. During the dissection, duplication of the gallbladder was encountered, which was an operative surprise as her ultrasound and CT scan did not reveal any evidence of duplication of the gallbladder (Figures 3–5). Both the gallbladders were lined by a single peritoneal lining, and it was only after proper dissection that the duplication was identified. Both the gallbladders were fused along their entire length. They both had their cystic duct entering their infundibulum. Both the gallbladders as well as their cystic ducts were dissected carefully. To better understand the anatomy, an intraoperative cholangiogram was performed (Figure 6). It seemed like both the cystic ducts joined proximally and formed a common cystic duct before draining into the common bile duct. Both the gallbladders were dissected and removed laparoscopically. The patient tolerated the procedure well and did well postoperatively. Her pathology revealed two separate gallbladders with some sludge and features of chronic cholecystitis.

## 3. Discussion

Gallbladder duplication is a rare congenital malformation seen in around one in 3800-4000 births. The exact incidence cannot be ascertained as most cases are detected at the autopsy, surgery, or imaging [1, 2].

The exact etiopathogenesis of gallbladder duplication is not clear; however, in 1926, Boyden reported that numerous outgrowths and accessory vesicles are formed during the 5th to 6th week of embryogenesis from a ductal system called the hepatic antrum. Usually, these supernumerary buds would regress, but the persistence of one of these buds would result in the formation of duplication of the gallbladder [3]. The other theories are splitting of cystic primordium and accessory cystic primordium [4]. In the *splitting of cystic primordium theory*, the duplication occurs due to the cystic primordium's bifurcation. A disturbance of cell division at the rapidly growing tip results in two tips, each of which is competent to form a complete, normal gallbladder [4]. In *double cystic primordia*, also called accessory gallbladder, two cystic primordia might arise separately from the common bile duct resulting in duplication of the gallbladder [4].

**Figure 7:**
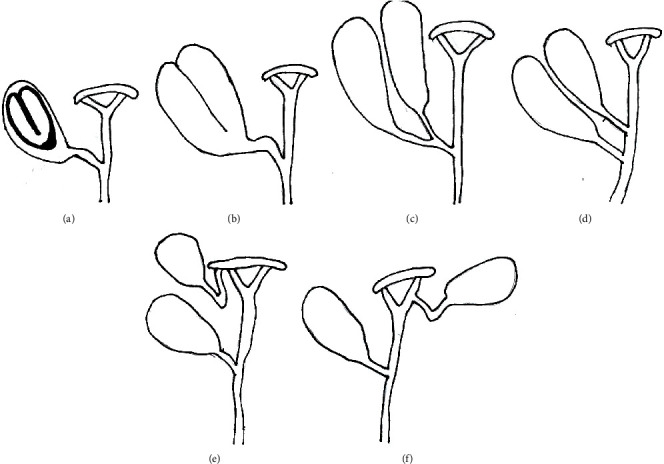
Classification of the duplication of the gallbladder.

Duplication of the gallbladder is classified based on the configuration of cystic ducts (Table 1) (Figure 7). The classification by Harlaftis et al. is the most accepted one, which categorized gallbladder duplication into type 1 (split primordium gallbladder) and type 2 (accessory gallbladder) with the respective incidence of 45.1% and 54.9% [4]. In the modified Harlaftis classification, one more variant is added where the accessory gallbladder drains into the left hepatic duct [5].

Clinically, patients with gallbladder duplication do not have any specific symptoms related to the duplication. Desolneux et al. reported that the incidence of developing a biliary disease is no different in patients with duplicated gallbladder than the ones with a single gallbladder [6]. Pillay et al., however, reported that the congenital malformations of the gallbladder could lead to a higher incidence of cholelithiasis due to inadequate drainage of bile [1].

Ultrasound of the gallbladder is a useful diagnostic tool for gallbladder diseases; however, the ultrasound has limited diagnostic value for identifying anomalies of cystic ducts and bile ducts [2, 6]. Magnetic resonance cholangiopancreatography (MRCP) is a much better diagnostic imaging in detecting the anatomic malformations of the gallbladder and biliary system [2, 6]. Endoscopic retrograde cholangiopancreatography (ERCP) and percutaneous transhepatic cholangiography (PTC) can reveal the anomaly, but both are invasive procedures and should be avoided for the diagnostic workup of duplication of the gallbladder in favor of noninvasive MRCP [7].

According to the available literature, it is not very clear if gallbladder duplication is associated with other congenital anomalies. According to Goh et al., no published literature had reported an association between a duplicated gallbladder and other duplex structures [8]. However, Szczech et al. and Udelsman and Sugarbaker have reported gallbladder duplication with biliary duct malformation or aberrant hepatic duct [9, 10]. Therefore, defining the exact anatomy of the biliary tree during surgery is crucial to reduce the risks of complications from the biliary and vascular injury, including damage to the common bile duct or other important nearby structures. In 2016, Gupta et al. reported two type 2 gallbladder duplication cases in the neonates, which coexisted with other gastrointestinal congenital malformations. One of the neonates had duodenal atresia, and the other neonate had pyloric, ileal, and colonic atresia [11].

Prophylactic cholecystectomy is not recommended for an incidentally found duplicated gallbladder. For a symptomatic gallbladder, laparoscopic cholecystectomy is the treatment of choice [1, 2]. Congenital malformations of the biliary system are considered the important predisposing factors for iatrogenic bile duct injury during cholecystectomy. Therefore, the identification of each infundibular-cystic duct junction is necessary during cholecystectomy [1, 2]. In the circumstances where an unexpected biliary anomaly is found intraoperatively, it is crucial to perform an intraoperative cholangiogram to complete a safe cholecystectomy [2].

Duplication of the gallbladder is quite rare, with only 56 cases reported in the literature so far (Table 2). In 1926, Boyden published a compilation of 20 cases of gallbladder duplication [3]. Slaughter and Trout reported a compilation of another 12 cases in 1933, and Weiss et al. reported three more cases of the double gallbladder in 1935 [12, 13]. Gross reported another case of the duplicated gallbladder in 1936, and another case was reported by Wilson in 1939 [14, 15]. Therefore, with 20 cases reported by Boyden, 12 reported by Slaughter and Trout, three by Weiss, one by Gross, and another one by Wilson, 37 cases of gallbladder duplication were reported until 1939 [15]. Since 1939, 19 more cases of gallbladder duplication have been reported (Table 2). The present case was a surprise finding at the time of surgery as the patient's preoperative workup, including ultrasound and CT scan, did not reveal any evidence of duplication of the gallbladder.

## 4. Conclusion

Duplication of the gallbladder is a rare congenital anomaly, which could be associated with other anomalies of the bile duct and vascular systems. Surgeons should be wary of this anomaly during the patient's preoperative workup and surgery as congenital anomalies are considered critical predisposing factors for iatrogenic bile duct injuries during cholecystectomy.

## Figures and Tables

**Table 1:** Classification of duplication of the gallbladder (Figure 7) [4, 5].

Type 1—one cystic duct entering the common bile duct	(i) Gallbladder septum (Figure 7(a)) (ii) Two gallbladders joining at the neck to form a single cystic duct (V duplication) (Figure 7(b)) (iii) Complete separation of two gallbladders, each with its cystic duct, which would join to form a common cystic duct (Y duplication) (Figure 7(c))
Type 2—two cystic ducts entering bile duct separately	(i) Two cystic ducts entering the common bile duct separately (H duplication) (Figure 7(d)) (ii) One cystic duct entering the common bile duct, and the other one entering the right or left hepatic duct (Figure 7(e)) (iii) Bilateral gallbladders with separate cystic ducts (Figure 7(f))

**Table 2:** Summary of the duplication of gallbladder cases reported since 1926.

Authors	Year reported	Cases/patient characteristics	Type of duplication
Boyden [3]	1926	20 cases	
Slaughter and Trout [12]	1933	12 cases	
Weiss [13]	1935	3 cases	
Gross [14]	1936	3-year-old male	2
Wilson [15]	1939	55-year-old female	2
Granone [16]	1984	34-year-old female	1
Udelsman and Sugarbaker [10]	1985	60-year-old female	2
Haghighi et al. [17]	2000	68-year-old female	2
Valadez et al. [7]	2004	44-year-old male	1
Barut et al. [18]	2006	55-year-old female	1
Asbury [19]	2007	70-year-old male	1
Desolneux et al. [6]	2009	61-year-old male	1
Causey et al. [5]	2010	15-year-old female	1
Hassan et al. [20]	2012	83-year-old female	2
Shiba et al. [21]	2014	38-year-old female	1
Pillay [1]	2015	56-year-old male	1
Szczech et al. [9]	2015	26-year-old female	1
Goh et al. [8]	2015	28-year-old male	1
Gupta et al. [11]	2016	12-day-old male and 2-day-old male (two cases)	1
Rajapandian et al. [22]	2017	28-year-old male	1
Ghaderi et al. [23]	2018	38-year-old male	2
Romero et al. [24]	2018	50-year-old female	1
Boukoucha and Dhieb [2]	2020	58-year-old female	1
